# Glucose Metabolism and Innate Immune Responses in Influenza Virus Infection: Mechanistic Insights and Clinical Perspectives

**DOI:** 10.3390/cells15010047

**Published:** 2025-12-26

**Authors:** Kareem Awad, Nancy N. Shahin, Tarek K. Motawi, Maha Abdelhadi, Reham F. Barghash, Ahmed M. Awad, Laura Kakkola, Ilkka Julkunen

**Affiliations:** 1 Institute of Biomedicine, Faculty of Medicine, University of Turku, 20520 Turku, Finland; laura.kakkola@utu.fi (L.K.); ilkka.julkunen@utu.fi (I.J.); 2 Academy of Scientific Research & Technology-Short Term Applied Research & Technology Transfer (ASRT-STARS), Cairo 11516, Egypt; 3 Institute of Pharmaceutical and Drug Industries Research, National Research Centre, Giza 12622, Egypt; 4 Biochemistry Department, Faculty of Pharmacy, Cairo University, Kasr El-Aini, Cairo 11562, Egypt; nancy.shahin@pharma.cu.edu.eg (N.N.S.); tarek.motawi@pharma.cu.edu.eg (T.K.M.); 5 Internal Medicine Department, Institute of Medical Research and Clinical Studies, National Research Center, Giza 12622, Egypt; mahahadi39@yahoo.com; 6 Institute of Chemical Industries Research, National Research Centre, Giza 12622, Egypt; reham_fawzy@yahoo.com; 7 Research and Innovation Office, California State University Channel Islands, Camarillo, CA 93012, USA; ahmed.awad@csuci.edu; 8 Clinical Microbiology, Turku University Hospital, 20521 Turku, Finland; 9 InFLAMES Research Flagship, University of Turku, 20014 Turku, Finland

**Keywords:** influenza, immune-metabolism, glucose metabolism, host-pathogen interactions, therapeutic targets, kinases

## Abstract

This review article discusses glucose metabolic alterations affecting immune cell responses to influenza virus infection. It highlights possible relationships between essential metabolic targets and influenza replication dynamics in immune cells. Thus, kinases as essential regulators of glucose metabolism as well as critical immune mediators during this infection such as interferons, tumor necrosis factor-alpha and transforming growth factor beta have been illustrated. Mechanistic highlights are provided for both the Warburg effect, where glycolysis shifts to lactate production during influenza infection, and the PFK1/PFKFB3 enzyme complex as the rate-determining regulator of glycolysis whose activity increases during the course of influenza infection. The mechanisms of mammalian target of rapamycin (mTOR) signaling as a promotor of glycolysis and a regulator of inflammatory cytokine production are discussed across various immune cell types during infection. We conclude that modulation of the metabolic changes associated with immune responses plays an important role in disease progression, and that targeting metabolic checkpoints or kinases may offer promising avenues for future immunotherapy approaches for the treatment of influenza virus infection. We also emphasize the need for further research to develop a comprehensive biological model that clarifies host outcomes and the complex nature of immune-metabolic regulation and crosstalk.

## 1. Introduction

Influenza is a communicable viral disease characterized by acute respiratory illness that causes annual epidemics leading to 3–5 million severe cases within approximately 5–15% of the world’s population infected with the virus [1,2]. The surface of the influenza virus particle displays three key proteins: hemagglutinin (HA), neuraminidase (NA), and M2 protein [3]. These glycoproteins are essential for viral infection and determine the influenza virus type and subtype [3,4]. HA facilitates the virus’s attachment to the host cell, while NA initiates infection by cleaving sialic acid residues on the surface of the infected cell [3,4].

Individuals with influenza viral infections can develop a wide range of clinical features including fatigue, headache, fever, nasal congestion, cough, sore throat, myalgia and muscular pain. Severe cases may progress to sepsis, hyperinflammation, pneumonia, and hypoxemia, potentially leading to multi-organ failure. Gastrointestinal symptoms, such as nausea, vomiting, abdominal pain, and diarrhea, are frequently observed, depending on the virus, its variant and the affected population [5,6,7]. Severe cases of influenza-associated pneumonia can present with systemic symptoms such as reduced platelet counts, decreased serum albumin levels and elevated blood urea nitrogen levels [8].

Antiviral medications play a crucial role in both treating and preventing influenza, particularly in high-risk populations. As of 2025, four FDA-approved antiviral drugs are recommended for influenza treatment in the United States: oseltamivir, zanamivir, peramivir, and baloxavir marboxil [9]. While these antiviral drugs have shown efficacy in reducing the duration and severity of influenza symptoms, the development of viral resistance remains a concern [10,11]. Vaccination remains the cornerstone of influenza prevention and control strategies. Three types of vaccines are currently licensed globally: inactivated vaccines, live attenuated vaccines and recombinant HA vaccines [12,13]. Despite the availability of seasonal and pre-pandemic vaccines, the control of influenza remains suboptimal. The ever-present risk of a pandemic caused by a novel virus strain, to which most of the population lacks immunity, underscores the need for continued vigilance and research in vaccine development [14].

The broad clinical features of influenza infection, as well as the virus’s remarkable ability to evolve multiple variants that evade vaccines and therapeutics, suggest interactions between the influenza virus and complex host factors. These factors include metabolic and immune signaling pathways that reprogram the host response, many of which remain poorly elucidated [15,16,17,18]. Influenza viruses, as intracellular pathogens, rely heavily on the host's metabolic machinery to facilitate their replication. This process is highly demanding and requires significant energy and resources within a short timeframe [19,20]. Studies have shown that influenza viral infections induce distinct metabolic alterations in host cells, which vary depending on the dynamics of the infection and the virus strain. These changes reflect both the virus’s specific strategies to take advantage of the host metabolism and the host cell’s adaptive responses to counteract viral replication [19,20,21].

Influenza virus infection induces systemic metabolic reprogramming through alterations in specific cross-metabolic signaling, such as glucose uptake, as well as at the cellular level, including different types of immune cells and respiratory epithelial cells, the main targets of influenza [16,17,19,20]. These systemic and cellular interactions shape and drive the resulting severity of influenza infection, therefore offering a target for the development of effective antiviral therapies or vaccines, as well as a means to understand the breadth of the clinical picture of this infection [20,22,23].

In this review article, glucose metabolic alterations affecting immune cell responses to influenza virus infection will be discussed. Essential regulators of glucose metabolism and critical immune mediators will be elucidated to provide insights into potential novel therapeutic and diagnostic markers for influenza infection.

## 2. Glucose Metabolic Alterations During Influenza Viral Infection

Influenza virus relies extensively on host cellular metabolism to meet the energy and biosynthetic demands of replication, a process requiring rapid energy resources [19,20,21,22,23]. Infections trigger pathogen-specific metabolic reprogramming, with variations observed across virus types and infection stages [19,20]. Key alterations involve glycolysis and energy metabolism, as well as immune cell metabolic dynamics mediated by metabolic-immune crosstalk. This highlights the dual role of metabolism during influenza pathogenesis, while the virus manipulates host metabolic pathways for its replication, host cells adapt metabolic programs to limit infection [19,20,21,22,23]. Targeting virus-specific metabolic dependencies (e.g., glycolysis inhibitors) offers a promising therapeutic strategy that complements antiviral drugs.

### 2.1. Influenza Virus-Driven Glucose Metabolic Reprogramming in Immune Cells

Influenza virus drives metabolic reprogramming in immune cells. Strategic modulation of glycolysis has emerged as a promising approach to enhance immunotherapy by targeting immune cell metabolism [23]. Immune cells exhibit distinct and dynamic metabolic profiles. For instance, macrophages and dendritic cells (DCs) show reduced tricarboxylic acid (TCA) cycle activity. M1-polarized macrophages prioritize glycolysis, whereas M2-polarized macrophages depend on TCA cycle activity [17,24]. Monocyte-derived DCs display diminished oxidative phosphorylation (OXPHOS) [15], and the mechanistic target of rapamycin (mTOR) signaling pathway plays a pivotal role in regulating natural killer (NK) cell function [18]. These metabolic pathways are highly adaptable to physiological challenges, such as viral infections [24]. Although influenza viruses lack intrinsic metabolic machinery, they exploit host cell metabolism to redirect resources, such as glucose, toward viral replication [21,24]. Collectively, these insights highlight the potential of metabolic interventions to optimize immune cell efficacy in therapeutic contexts.

The Warburg effect has been reported during influenza virus infection, as cells exhibit a higher rate of glycolysis, rather than relying on the more cost-effective TCA cycle for the generation of energy [20,24]. The TCA cycle is not totally inactive; rather, it operates at a lower rate than in normal conditions, resulting in increased lactate levels [20,21,24]. Viral and cellular oncogenes converge on targeting networks of critical proteins to reprogram the cellular DNA replication and protein synthesis machinery, leading to pathological outcomes [20,24]. Moreover, this metabolic shift may promote the survival of infected cells, providing an ultimate advantage to the virus [18]. Influenza virus-induced metabolic reprogramming is highly cell-specific and depends on the capability of the virus to adapt to the unique host cellular environment [19,20,21].

It has been previously shown that influenza virus infection has its main impact on host metabolism during the late stages of the replication process by modulating phosphofructokinase 1 (PFK1) and the product of glycolysis, pyruvate [21]. Changes in the rate of glycolytic activity are primarily driven by the upregulation of key glycolytic enzymes, including hexokinase, PFK1 and pyruvate kinase [20,25]. In contrast to glycolysis, most intermediates in the TCA cycle and associated metabolic pathways are significantly reduced following influenza infection, suggesting a reduced substrate supply to the TCA cycle under conditions of differential enzymatic activities [26,27]. Among the metabolic enzymes that also modulate glycolysis are the 6-phosphofructo-2-kinase/fructose-2,6-bisphosphatases (PFKFBs), a family of bifunctional enzymes that control the levels of fructose-2,6-bisphosphate (Fru-2,6-P2), an essential activator of the glycolytic flux [28,29]. Fru-2,6-P2 potently activates glucose breakdown in glycolysis through allosteric modulation of the rate-limiting enzyme of glycolysis, PFK-1 [28,29,30]. Accordingly, a correlation might be expected between aberrant PFKFB expression level and the grade of infection. Positive or negative statistical correlations have been reported between PFK1 and specific immune mediators, such as TNFα, TLR3 and TLR7 at the protein or gene level in cultured monocytes upon influenza infection [19].

### 2.2. Immune Cell Adaptation to Metabolic Signaling

Immune cells can adapt to these induced metabolic shifts by secreting regulatory factors that function through different mechanisms. Numerous studies have shown that interferons (IFNs) are potent modulators of fundamental cellular processes [31,32]. Type I IFNs promote glycolysis by stimulating phosphatidylinositol-3-kinase/protein kinase B/mTOR (PI3K/AKT/mTOR)-dependent glucose uptake, suggesting an antiviral state and a role for the PI3K/AKT signaling pathway during influenza virus propagation in immune cells [31,32,33,34,35]. Similarly, activation of macrophages by type II IFN induces elevated glycolysis and a disruption of TCA flux [31,32], leading to the accumulation of succinate and citrate [34]. The latter drives mitochondrial reactive oxygen species (ROS) production, a conserved response against many pathogens that induces apoptosis of infected cells [31,34,35,36].

In macrophages, itaconate reduces ROS levels and STAT1 phosphorylation, resulting in reduced interferon responses and inflammation during influenza A virus infection [37]. Another mechanism in macrophages involves the signaling adaptor MAVS, which regulates how hexosamine biosynthesis pathway (HBP)-associated O-linked β-N-acetylglucosamine (O-GlcNAc) signaling promotes antiviral innate immunity [38]. This highlights an important modulation of glucose metabolism as an antiviral innate strategy [37].

NK cells utilize mTOR signaling to promote their maturation. This mTOR signaling pathway is downregulated to enforce quiescence in mature NK cells. In response to viral infection, NK cells activate mTOR signaling to enable expansion and sustain oxidative metabolism, thereby exiting the quiescence status [39].

Recent studies have highlighted the important role of hypoxia-inducible factor 1-alpha (HIF-1α) in influenza-induced immune modulation, regulation of inflammatory responses, and virus pathogenesis, proposing it as a potential target for strategic intervention in influenza infection [40].

Metabolic switching in DCs plays a vital role in immune modulation during influenza infection. In DCs, c-Myc activity restriction significantly alters immune functions; thus, early in the infection process, DCs undergo broad metabolic changes that are vital for effector function [41]. This transcriptional modulation may impair functions related to T cell priming and optimal DC motility, thereby reducing the capacity of the T cell response to infection [41]. A strategy for manipulating PFK1-related metabolism has been demonstrated in innate immune cells through interferon regulatory factor-7 (IRF7)- and NF-κB-mediated signaling, thus enhancing IFN-β antiviral activity [42]. The same study also reported another unique modulatory mechanism where arrestin domain-containing 4 (ARRDC4) senses influenza infection and regulates innate immunity through the muscle variant of the PFK1 metabolic axis, leading to antiviral activity [42].

### 2.3. Clinical Insights into Glucose-Dependent Immune Metabolic Reprogramming 

Hyperglycemia orchestrates immune cell dysfunction during respiratory viral infection and drives a metabolic–immune axis that may be therapeutically exploited to attenuate exacerbated responses in hyperglycemic infected patients [43]. Diabetes represents a major global burden and is a well-established risk factor for increased severity of influenza virus infection. However, the mechanisms by which high glucose levels exacerbate influenza virus infection severity are not yet well declared, leaving patients with diabetes at a life-threatening susceptibility to influenza infection [43].

In diabetic patients, hyperglycemia impairs CD8+ T cell responses to influenza virus infection, as increased HbA1c levels correlate with reduced TNF-α production by these cells in response to influenza stimulation [44]. This is not associated with any changes in CD8+ T cell subsets and may be linked with the increased risk of severe influenza in individuals with diabetes [44]. High-glucose conditions prior to influenza infection exacerbate influenza-induced damage to the epithelial–endothelial complex and are associated with an augmented pro-inflammatory response [45]. This suggests that hyperglycemia may damage the pulmonary epithelial–endothelial barrier, promoting pulmonary edema and thereby increasing influenza severity. Thus, maintaining long-term glycemic control in diabetic patients is a critical factor in reducing influenza-associated morbidity and mortality [45].

### 2.4. Pharmacological Targeting of Glucose-Dependent Metabolic Reprogramming

Influenza A virus induces the glycolytic pathway in order to facilitate efficient viral replication, suggesting the possibility that glycolytic inhibitors can be used to treat influenza A virus infection as a future direction [46,47,48,49]. As demonstrated (Table 1), complex glucose metabolic pathways are regulated during influenza infection and may serve as attractive targets for the development of novel therapies.

**Figure 1:**
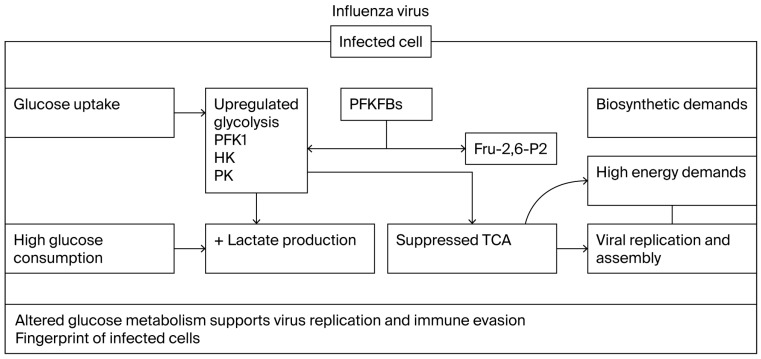
Kinases as possible targets during influenza viral infection. Metabolic pathways in immune cells are modulated either through interactions with metabolic signals from the affected organs such as diverse conditions of nutrients and oxygen availability or co-effectors in their macro- and micro-environment, e.g., infection, infection products or chemicals. The need for energy for such cellular functions is essential, with glucose metabolism being the major carbon fuel source through two distinct pathways: glycolysis as a first path producing pyruvate and ATP and the tricarboxylic acid cycle as an immediate following path. Essential kinases of interest include rate-limiting enzymes such as hexokinase (HK), phosphofructokinases 1 (PFK1), pyruvate kinase (PK) and 6-phosphofructo-2-kinase/fructose-2,6-bisphosphatases (PFKFBs).

In a broad context, kinases are ideal candidates for monitoring and intervention, as they regulate critical cellular processes exploited by most viruses. Influenza infection alters host metabolic processes, with most of these alterations mediated by kinases. These kinase-driven modifications influence influenza replication, infection kinetics and pathogenicity [50]. Since the majority of host cell alterations following influenza infection occur within metabolic pathways, influenza-regulated kinase activity can be expected to have a major influence on reprogramming cellular metabolism (Figure 1; [51]). A notable example of a kinase involved in critical cellular processes is Bruton’s tyrosine kinase (Btk), which is expressed in B-cells and has a role in B-cell antigen receptor signaling [52]. Genetic defects in Btk lead to Bruton syndrome, the failure of B-cells to produce antibodies [53]. Inhibition of the Btk gene induces metabolic stress through suppression of PI3K/AKT/mTOR signaling, highlighting the link between cellular metabolism and innate immunity [54]. Interestingly, using a PI3K/mTOR inhibitor to disrupt glucose metabolism in vitro resulted in reduced influenza virus production [51].

**Table 1:** A summary of immune cell modulations during influenza infection [40,41,47,48,49].

Immune Cell Type		Metabolic Profile	Metabolic Mechanism	Influenza Alteration
Macrophages [20,27,48]	M1	Reduced TCA cycle Increased glycolysis [19,20,27]	PI3K/mTOR inhibition [33,49,51] HIF-1α stabilization [31,40] Induction of resolution [47] GAPDH inhibition [48] c-Myc involvement [41]	Dominated glycolysis “Warburg effect” [20,48]
	M2	Reduced TCA cycle Reduced glycolysis [20,27]		Dominated glycolysis [20,48]
Dendritic cells [41]		Diminished OXPHOS [41,48]		Suppressed OXPHOS Altered glycolysis [41,48]
Natural killer cells [39,48,49]		Active mTOR [39,48,49]		Inhibited mTOR [39,48,49]
T-cells/B-cells [40,48,49]		Normal glycolysis [48]		Inhibited glycolysis Increased OXPHOS [40,49]

More than 500 human kinases have been identified and are categorized based on their phosphorylation substrates as tyrosine, serine/threonine or lipid kinases [55]. Therefore, a more comprehensive understanding of how influenza viruses utilize these critical host factors, and how these kinases regulate species-specific host adaptation and influenza virus pathogenesis, is required and could hopefully lead to more effective treatment strategies [50]. 

## 3. Cellular Immune Responses During Influenza Viral Infection

**Figure 2:**
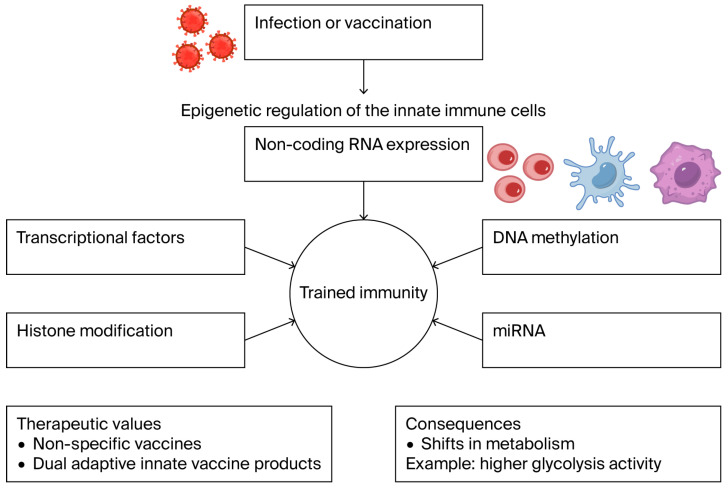
The concept of trained immunity/immune reprogramming. Changes in innate immune cells can be achieved by epigenetic manipulation rather than permanent genetic modification. The production of dual innate adaptive vaccine products proposes a protective value for epigenetic changes and proposes a therapeutic potential when considering the associated kinases responding to the alterations in cellular energy.

The immune system has evolved to protect against pathogenic microbes while avoiding damage to self-tissues. The discovery that the adult immune system can tolerate non-immunogenic forms of antigens was fundamental in shaping current approaches to establish immune tolerance and reprogram the immune system for therapeutic purposes. Reprogramming of the immune system can be achieved through different mechanisms beyond permanent genetic changes [56]. In particular, long-term changes in innate immune cells can be attained by epigenetic manipulation of cell physiology and transcriptional regulators rather than permanent genetic modification. Such long-term changes enhance responsiveness upon second stimulation by microbial infection. These adaptations hold therapeutic potential for the development of non-specific vaccines or dual adaptive innate vaccine strategies. However, the associated alterations in cellular energy and hyperactive metabolic status can lead to deleterious effects. Innate trained immunity has been observed in human monocytes and macrophages following influenza infection, characterized by a shift from OXPHOS to aerobic glycolysis, and mTOR/AKT-driven metabolic reprogramming ([57]; Figure 2).

Infected cells sense viral pathogen-associated molecular patterns (PAMPS) through cell-specific pattern recognition receptors (PRRs; [58]). Three families of PRRs are involved in sensing viral PAMPs: toll-like receptors (TLRs), retinoic acid-inducible gene I (RIG-I), and nucleotide oligomerization domain (NOD)-like receptors [59]. Stimulation of these receptors triggers the expression of IFNs, chemokines, and other immune orchestrating proteins. These immune mediators induce an antiviral state by inhibiting viral spread and recruiting specific immune cells for viral clearance [59]. Innate immunity plays an important role in the early phases of influenza virus infection. DCs and macrophages are two key innate immune cell types that reside in tissues where they serve to alert the immune system to pathogen invasion. DCs take up microbes or their components or may become directly infected by viruses. This leads to their activation, increased cytokine production and migration of activated DCs into the local lymph nodes where they present microbial antigens to T cells [58,59,60,61,62,63].

The influence of pre-immunity was first suggested in the 1960s when a first exposure to the virus influenced the clinical outcome of the following infection, a phenomenon that is pointed out as original antigenic sin (OAS; [64,65,66,67]). OAS was described as a failure to control the second viral infection because of the memory immune responses developed toward epitopes of the initial infecting virus [64]. However, the immune history is not merely composed of the host’s first influenza virus infection, but rather, to our current knowledge, it involves the dynamic interaction between the immune system, host metabolic factors, and each exposure to influenza viruses [56,63].

### 3.1. Inflammatory Cytokines

Cytokines are glycoproteins secreted by various types of cells. They are involved in host defense and inflammatory responses, contributing both to protection against pathogens and to disease pathogenesis [68,69]. The mechanisms of defense and pathogenesis vary depending on factors such as the type of pathogen, the stage of infection (acute or chronic), and the site of infection (systemic or localized [69]). Classical pro-inflammatory cytokines include transforming growth factor-β (TGF-β), tumor necrosis factor-alpha (TNF-α), and IFNs [70]. It is now clearly known that severe cytokine storms, with elevated levels of IFNs and TNF-α, have been observed in patients hospitalized due to influenza infection [71]. Such influenza-induced cytokine storms, together with high viral loads, can develop severe lung injury in patients and contribute to the severity of the disease [72,73,74,75]. Influenza viral infection induces the production of type I IFNs in immune cells, resulting in Krebs cycle flux disruption accompanied by citrate and succinate accumulation, alongside a high flow of glycolysis. This metabolic shift facilitates viral replication and suggests potential pathways to target therapeutically [20]. The crosstalk between influenza-induced cytokine production and metabolic pathways has been evidenced [76]. Influenza virus modifies mammalian target of rapamycin (mTOR) signaling to enhance glycolysis, supporting its replication. Beyond promoting aerobic glycolysis, mTOR regulates multiple cytokines, including TGF-β, type I IFN, and TNF-α [77]. Glyceraldehyde 3-phosphate dehydrogenase (GAPDH) is a key glycolytic enzyme coordinating cytokine production and glycolytic flux while limiting inflammatory cytokine gene expression through the GAIT complex [78]. Live or inactivated influenza induce innate responses in B lymphocytes via a myeloid differentiation primary response gene 88 (MyD88)-dependent pathway, suggesting the involvement of TLR family signaling in this interaction [79].

### 3.2. Interferons

**Figure 3:**
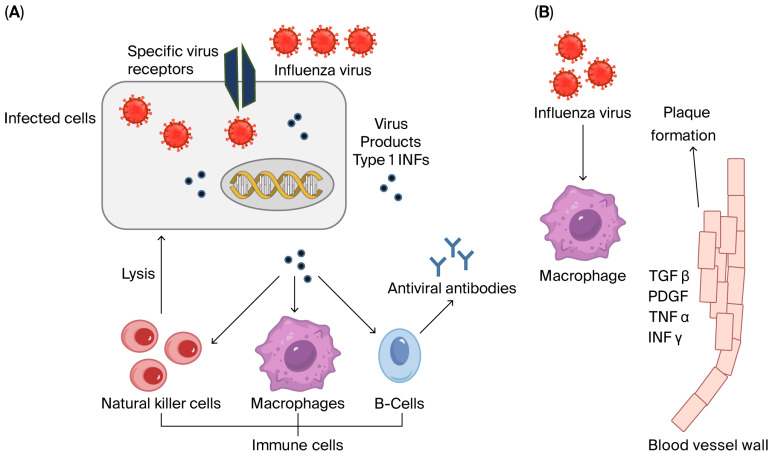
Human cell responses to influenza viral infection. Pathogens are detected through the recognition of specific molecular structures or surface receptors that are absent from uninfected cells, termed pathogen-associated molecular patterns (PAMPs). Two types of pattern recognition receptors (PRRs), which are toll-like receptors (TLRs) and retinoic acid inducible gene-I (RIG-I)-like receptors (RLRs), can signal infection and then activate immune cells to produce specific markers. Each PRR depends on the cell type affected and the nature of the pathogen. Viruses attack both immune and somatic cells. When they attack somatic cells (**A**), mainly type *I* interferons are produced by infected cells, which act on both innate and adaptive immune cells to introduce these infected cells to innate immune monocytes and macrophages and present them to naive B cells, which then produce virus-specific antibodies. On the other hand, when the virus attacks immune cells (**B**), specific inflammatory cytokines are released. These include TNF-α as well as growth factors, which may lead to epithelial hyperplasia or plaque formation. Multiplication of viruses, however, in immune cells is controversial. TGFβ: transforming growth factor beta; PDGF: platelet-derived growth factor; TNFα: tumor necrosis factor alpha; INFγ: interferon gamma.

Interferons (IFNs) are classified into three types according to their amino acid sequences and their receptor type. Type I IFNs, which include IFN-α subtypes and IFN-β, as well as type III IFNs (IFN-λ1-IFN-λ4), are rapidly upregulated following influenza infection. In contrast, type II IFN (IFN-γ), is secreted later by activated immune cells [80,81,82,83]. The production of type I IFNs (IFN-α/β) is a fundamental step in counteracting influenza viral infections [80,81]. Once a cell is attacked by virus particles, type I IFNs are produced. These IFNs are released from the infected cells and target innate and adaptive immune cells to engulf the infected cells and to produce specific antibodies, respectively (Figure 3; [80,81,82]). Multiple factors influence IFN induction by influenza virus during an infection, which include the rate of viral replication, the ability of the virus to actively antagonize IFN induction, and host-specific factors [80,81]. 

IFN induction signals influenza infection and triggers metabolic reprogramming of glucose metabolism to a status of enhanced glycolysis and reduced TCA cycle activity [19]. A significant inhibition of influenza-induced IFN-β mRNA expression has been observed under high-glucose culture conditions compared to normal glucose culture. This was related to either increased lactate formation and PFK overactivity or to the direct effect of high cellular glucose concentrations, leading to the reduced expression of viral RNAs, reducing the ability of the infection to stimulate the RIG-I pathway and IFN-β gene expression [81,83]. 

Glucose-regulated protein 78 (GRP78) can exert crucial functions during influenza virus infection by inhibiting influenza replication, through the activation of STAT1/2 signaling and the induction of antiviral IFN-stimulated genes [84]. Clinical analysis revealed an association between interferon regulatory factor-5 (IRF5) and the hexosamine biosynthesis pathway (HBP)-associated O-linked β-N-acetylglucosamine (O-GlcNAc), where higher levels of pro-inflammatory cytokines correlate with elevated levels of blood glucose in influenza-infected patients. This highlights a molecular mechanism by which HBP-mediated O-GlcNAcylation regulates IRF5 function during influenza infection and underscores the vital role of glucose metabolism in influenza-induced cytokine storm [85].

IFN production following viral infection results in significant alterations in cellular glucose metabolism, and understanding these changes is essential, as insights into influenza virus replication have elucidated novel therapeutic avenues that rely on targeted inhibition of such cellular metabolic pathways [86]. One such mechanism involves histone H1.2-mediated promotion of IFN-β production by targeting the melanoma differentiation-associated protein 5 (MDA5) signaling, thereby modulating influenza virus replication [87]. A deeper understanding of the mechanisms controlling the interactions between the influenza virus and the IFN system could enhance the development of novel anti-influenza therapies.

### 3.3. Tumor Necrosis Factor-α (TNF-α)

Tumor necrosis factor-α (TNF-α) has shown early and late upregulation in monocyte-derived DCs and primary human macrophages upon infection with the highly pathogenic H5N1 and the less pathogenic H7N9 influenza viruses, respectively [88,89]. TNF-α enhances influenza virus-induced expression of antiviral cytokines by activating retinoic acid inducible gene-I (RIG-I) expression [90]. A significant correlation was evidenced between TNF-α protein and mRNA expression and the glycolysis rate-limiting enzyme PFK following influenza infection of monocytes [19]. As discussed above, increased HbA1c levels correlate with reduced TNF-α production by CD8+ T cells in response to influenza stimulation, linking this with the increased risk of severe influenza in patients with diabetes [44]. TNF-α induces reactive oxygen species (ROS) and nitric oxide (NO) production in immune cells, consequently stabilizing hypoxia-inducible factor 1 alpha (HIF-1α), which translocates to the nucleus, driving the expression of glycolysis-activating genes, predominating lactate production over the pyruvate pathway [20,40,91].

### 3.4. Transforming Growth Factor Beta 1 (TGF-β1)

Transforming growth factor-beta 1 (TGF-β1) belongs to a family of small proteins that perform diverse functions in immunological, physiological, and cellular processes through seven main Smad proteins whose signaling pathways diverge and converge with almost all other known signaling networks [92,93,94]. Influenza virus infection results in increased secretion of cytokines involved in acute inflammatory stress at sites of viral replication, suggesting the contribution of specific secreted transcription factors to the pathogenesis of influenza virus [95,96,97,98]. 

Pulmonary epithelial cells, which play a central role in orchestrating the defense against respiratory pathogens, produce TGF-β to suppress early immune responses during influenza A infection. In this context, epithelial-derived TGF-β can act to suppress early IFN responses, potentially aggravating the consequences of the viral infection [81,99]. Both influenza A and B viruses can trigger epithelial–mesenchymal transition (EMT) as a mechanism of lung tissue repair following injury. This mechanism is known to be mediated by the TGF-β/Smad2 signaling pathway, depending on the binding ability of latent TGF-β to viral NA [100].

In human macrophages, TGF-β1 has been demonstrated to correlate with increased lactate levels and significantly increased PFK activity in high-glucose concentration, leading to specific Smad phosphorylation patterns (Smad2/3 or Smad1/5). Such metabolic status is similar to that observed in influenza-infected lung epithelial cells under high-glucose conditions [81,94]. This suggests that TGF-β1-Smad signaling may serve as a specific metabolic marker of influenza infection, opening an interesting research area. As a potential diagnostic biomarker, TGF-β serum levels have been shown to distinguish H1N1 pdm09 infection from other causes of pneumonia that develop sepsis [101].

### 3.5. Toll-like Receptors (TLRs)

The IFN induction cascade is triggered following the recognition of certain molecular structures that are absent in uninfected cells, termed pathogen-associated molecular patterns (PAMPs; [58,59]). For influenza and other RNA virus infections, these PAMPs are predominantly distinct features of viral RNA that are not typically present in cellular RNAs, such as regions of double-stranded RNA (dsRNA) or the presence of 5′-triphosphate or 5′-diphosphate groups [102,103,104]. Pattern-recognition receptors or pathogen-recognition receptors (PRRs) play a critical role in distinguishing self from non-self-molecules by binding to PAMPs within infected cells [102,103,104]. 

Influenza virus is a negative single-stranded RNA (ssRNA) virus that is recognized, in particular, by TLR7, which was verified to be expressed in macrophages and DCs [105,106]. TLR3 is also activated by influenza virus infection [107]. The mTOR-associated pathway involving AMP-activated protein kinase (AMPK) is a key mechanism underlying influenza-induced metabolic reprogramming in TLR 7/9-activated-plasmacytoid dendritic cells (pDCs). This mechanism promotes IFN-α production and is strongly implicated as a driver of metabolic reprogramming in immune cells [108]. mTOR also interacts with the TLR7 signal transducer, MyD88, to activate IFN regulatory factors 5 and 7 (IRF5 and IRF7) and promote cytokine production [109]. However, influenza infection of DCs can lead to a metabolic phenotype distinct from that induced by TLR signaling, through the c-Myc transcription factor (41). These observations suggest that mTOR and its associated kinases represent potential antiviral targets that warrant consideration as future therapeutic strategies [110]. 

Organisms have evolved mechanisms for modulating their TLR-mediated responses to limit exaggerated innate responses that could lead to harmful outcomes [111].Such regulatory mechanisms include the production of members of interleukin-1 receptor-associated kinase (IRAK) family of serine/threonine kinases or suppressor of cytokine signaling (SOCS) family of proteins that negatively regulate cytokine signaling pathways, the release of specific membrane-bound proteins that negatively regulate TLR signaling, the action of specific ubiquitination ligases, or a combination of these negative regulators [111].

## 4. Current Perspectives and Suggested Future Research Directions

### 4.1. Targeting Metabolic Checkpoints

Metabolic pathways have always been known for their complexity involving sophisticated regulatory enzymes and intracellular metabolites that control them [112]. Immunometabolism currently presents the core interest of both immunologists and biochemists, highlighting new discoveries regarding the role of metabolic pathways in immune cell function [113]. It is now evident that signals from the immune system can regulate the host metabolite availability and metabolic pathways, thereby inducing changes in cell polarization and function. However, unresolved questions in this research area are still demanding future research effort [113]. 

Comparing the metabolism of resident macrophages from diseased and healthy tissues, as well as from different tissues under resting conditions, has shown quite divergent gene expression profiles [113,114]. In our previous studies on U937 cells, we reported a statistical correlation between glycolytic and inflammatory changes at both the transcriptional and translational levels following infection with the PR8-H1N1 virus. Further investigation into the epigenetic mechanisms underlying this correlation is recommended [19,25]. Much remains to be understood about the interplay between metabolic pathways, epigenetic control of gene expression and the regulation of transcription factors, either through ubiquitination mechanisms or negative feedback in macrophages and other immune cells. It can be suggested that manipulation of these pathways may change the function of immune cells in specific ways, beyond their traditional roles in energy generation and general biosynthesis [112]. Thus, metabolic reprogramming of immune cells may be driven by immunoregulatory elements that govern the nature of the immune response in both healthy and diseased conditions, where modulation or inhibition of glycolysis or glycolytic kinases, whether by therapeutic intervention or pathogen-derived regulatory immune elements, represents an attractive target.

### 4.2. Kinases as Dual Metabolic–Immune Regulators

Kinases represent strong candidates for antiviral therapies by linking critical cellular processes utilized by most viruses. Their involvement in pathological conditions has led to the development of inhibitor molecules and the repurposing of clinically approved drugs to treat infectious diseases such as influenza [19,20,114]. Investigating the potential immunomodulatory effects of kinases during influenza infection will enhance our understanding of their role in viral pathogenesis and may lead to novel intervention strategies [19,20,25]. Further research into the contribution of host kinases to influenza-induced metabolic changes is in progress and provides the impetus for additional avenues of basic and translational research [114]. 

It could be assumed that therapies targeting host cell factors would effectively reduce disease severity and improve patients’ recovery. In the U937 model of influenza infection, it has been clearly demonstrated that the glycolytic enzymes PFK1 and PFK2 are significantly and differentially expressed at both the protein and gene levels based on prior exposure to viral antigens or to the chemical stimulus phorbol myristate acetate (PMA; [19]). This observation may suggest that monitoring the host immune cell’s status and exposure history should accompany strategies aimed at targeting host kinases. 

The realization that polarized macrophages assign substantial resources to produce itaconic acid, and that succinate plays important roles beyond its classical function as a TCA cycle intermediate, raises important questions regarding the roles of these metabolic intermediates in the regulation of immune cell function [115,116,117].

From another perspective, host kinases may exist in multiple isoforms with different functionalities [19,25]. This has been illustrated in the context of PFK2. PFK2 has several isoforms that have been shown to differentially regulate glycolysis based on the cell type, cellular state, or the surrounding effector molecules [118]. PFKFB3 is a PFK2 isoform encoded by the *pfkfb3* gene [30]. PFKFB3 regulates intracellular levels of the glycolytic intermediate fructose-2,6-bisphosphate (F-2,6-BP), which allosterically activates 6-phosphofructo-1-kinase, thereby increasing the glycolytic flux [119,120]. Upregulation of PFKFB3 expression is a critical step in the induction of glycolysis in M1-activated macrophages [118]. Upon stimulation by LPS, macrophages differentiate into the M1 phenotype, which increases the expression of specific cell surface receptors for adenosine [121]. The binding of adenosine to these receptors further increases LPS-induced expression of PFKFB3, leading to increased glycolytic activity [121]. On the other hand, M2 macrophages predominantly express PFKFB1, a different isoform of PFK2 [30]. PFKFB1 exhibits higher bisphosphatase activity than PFKFB3 [119,120] and therefore more readily breaks down F-2,6-BP into fructose-6-phosphate, decreasing glycolytic activity [118]. In our previously published work, we demonstrated that using shared primers for the five known isoforms of PFK2 in the U937 human monocyte model revealed significant differences in the gene expression levels following H1N1 influenza infection. These differences were observed based on the initial cellular stimulus, either with the virus or PMA, which suggested the need for further investigation of the expressed isoform following macrophage stimulation or infection [19]. 

### 4.3. Integrating Metabolomics and Immunotherapy in Influenza Research

Innate responses have demonstrated remarkable specificity and complexity, as shown through mediators of the TLR family [19,122,123]. In particular, TLR3 and TLR7, among other cytoplasmic sensors of RNA, are critical for activating these responses [19]. The dual nature of innate immunity highlights an inherent paradox, as some aspects of the innate responses may contribute to morbidity and mortality, while the useful outcome could be that the innate responses restrict viral replication and foster effective adaptive responses, thereby preventing future infections by the same viral lineage [122,123]. Puzzling questions related to influenza, such as why some patients develop severe infections while others experience self-limiting responses, underscore the need for further research to clarify such regulatory mechanisms controlling these divergent outcomes. One example of such mechanisms involves alveolar macrophages, which play a key role in engulfing infected cells, releasing pro-inflammatory chemokines and cytokines, and orchestrating early host responses to influenza [124,125]. During influenza virus infection, monocytes and macrophages exert protective functions that may be exploited to develop strategies for modulating immune signals derived from these cells.

In the context of influenza virus infection severity, cytokines released by immune cells can play a paradoxical role, displaying both beneficial and deleterious effects. For instance, TLR4 activation triggered by H5N1 influenza virus leads to acute lung injury [126]. Moreover, in TLR3-deficient mice, the course of influenza virus infection was ameliorated [127]. Conversely, inhibition of macrophage responses in pigs was shown to be harmful [128]. Thus, it is fundamental to distinguish between detrimental and protective specific innate immune responses. It remains of critical importance to determine whether inhibition of specific aspects of the immune response could benefit severe influenza cases.

Our previous work, alongside that of others, suggests that our understanding of the complex interactions among innate immune cells remains undefined, warranting further investigation [20,25,29,129]. Designing innate immune inhibitors can offer promising therapeutic approaches. Another ongoing challenge is to develop measurable biological models that closely resemble human influenza infection, in order to determine which aspects of the innate response can be safely modulated without compromising antiviral activity, an understanding that could help develop improved therapies or vaccine strategies for influenza [130,131,132]. This is particularly relevant for generating non-clinical data to substantiate clinical observations and provide insights into vaccine-induced protective immunity and its underlying mechanisms [133,134]. Harnessing biological systems in this way can help develop improved treatments and vaccine strategies for influenza in the future.

## 5. Conclusions

**Figure 4:**
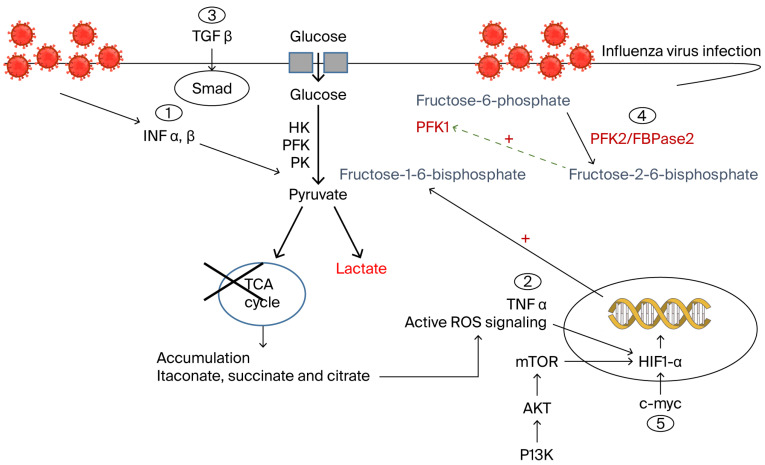
Cytokine–metabolism interactions during influenza virus infection of immune cells: Three inflammatory cytokines are highlighted in this work. (1) Type I IFN signals influenza infection, resulting in increased glycolysis and disruption of the TCA cycle. This mechanism involves stimulation of phosphatidylinositol-3-kinase/protein kinase B/mTOR (PI3K/AKT/mTOR)-dependent glucose uptake and disruption of TCA flux; (2) TNF-α induces ROS and nitric oxide production, stabilizing HIF-1α, that translocates to the nucleus, inducing the expression of glycolysis-activating genes, resulting in predominant lactate accumulation; (3)TGF-β1 is associated with increased lactate levels and PFK activity, establishing itself as a Smad-specific metabolic marker during influenza infection; (4) Among the metabolic enzymes that modulate glycolysis is PFK2 (PFK2/FBPase2), a family of bifunctional enzymes that control the levels of fructose-2,6-bisphosphate. PFK2 allosterically modulates the rate-limiting glycolytic enzyme PFK-1. Accordingly, a correlation might be expected between PFK1 and PFK2 expression levels and the grade of infection; (5) DCs can respond to influenza through a distinct metabolic phenotype, different from that induced by Toll-like receptor signaling, mediated by the transcription factor c-Myc. This metabolic reprogramming may impair DC functions related to T cell priming and optimal motility, thereby reducing DC migration and diminishing the capacity of the T cell response to infection. IFN: interferon; TNFα: tumor necrosis factor alpha; mTOR: mechanistic target of rapamycin; ROS: reactive oxygen species; HIF-1α: hypoxia-inducible factor 1 alpha; TGF β1: transforming growth factor β1; PFK: Phosphofructokinase; DCs: dendritic cells.

Controlling immune responses plays a pivotal role and may inform future immunotherapeutic approaches for the treatment of viral infectious diseases, particularly influenza. Long-term therapeutic benefits can be achieved by training immune cells through metabolic and epigenetic reprogramming, thereby enhancing their function and promoting a more effective immune response to secondary stimuli. Essential metabolic pathways drive immune cell activation and differentiation, while metabolic substrates, inflammatory cytokines, and other regulatory factors, such as kinases, regulate these pathways (Figure 4). The functionality of immune cells depends on the infection status and glycolytic activity that could either enhance or diminish their performance. Glucose concentrations in infected pulmonary cells change over time following influenza infection, at least as evidenced in vitro [83,135]. Influenza replication relies on glycolysis and glucose metabolism; however, blocking glycolytic kinases during influenza infection may result in adverse host consequences. Yet, kinases remain attractive targets for modulating glycolysis, and thus, regulating their activity may enable control of influenza pathogenesis. Overall, immunometabolism and metabolism-targeted interventions represent promising future directions for therapeutic development.

## Data Availability

Not applicable.
